# Taking a partnership approach to embed physical activity in local policy and practice: a Bradford District case study

**DOI:** 10.1186/s12966-024-01704-5

**Published:** 2025-01-07

**Authors:** Jennifer Hall, Elliot Lever, Nathan Dawkins, Emma Young, Jamie Crowther, Rachel Williams, John Pickavance, Sally Barber, Andy Daly-Smith, Anna Chalkley, On behalf of the wider JU:MP team

**Affiliations:** 1 Bradford Institute for Health Research, Bradford Teaching Hospitals NHS Foundation Trust, Bradford Royal Infirmary, Duckworth Lane, Bradford, BD9 6RJ UK; 2 Bradford Centre for Qualitative Research, Bradford Teaching Hospitals NHS Foundation Trust, Bradford Royal Infirmary, Duckworth Lane, Bradford, BD9 6RJ UK; 3 Faculty of Health Studies, University of Bradford, Richmond Road, Bradford, BD7 1DP UK; 4 Wolfson Centre for Applied Health Research, Bradford Teaching Hospitals NHS Foundation Trust, Bradford Royal Infirmary, Duckworth Lane, Bradford, BD9 6RJ UK; 5 School of Psychology, University of Leeds, Leeds, LS2 9JT UK; 6 Bradford Centre for Health Data Sciences, Bradford Teaching Hospitals NHS Foundation Trust, Bradford Royal Infirmary, Duckworth Lane, Bradford, BD9 6RJ UK

**Keywords:** Policy, Strategy, Sector, Whole-system, Co-production, Collaboration, Mixed-methods

## Abstract

**Background:**

Supportive policy is an important component of a whole-systems approach to increasing physical activity and reducing inequalities. There is a growing body of literature surrounding the design and effectiveness of national policy approaches to physical activity, but evidence related to local-level approaches is lacking. The aim of this study was to examine ‘what works’, and identify factors underpinning change, focused on work to embed physical activity in local policy and practice in Bradford, UK.

**Methods:**

A mixed-methods case study approach involved collecting data from cross-sectoral stakeholders directly or indirectly engaged in the physical activity agenda in Bradford over a period of three years (2021–2024). Data collection included focus groups, semi-structured interviews, researcher observations of key workshops and meetings, and surveys at two time-points (December 2021 and January 2024). Qualitative data were analysed using reflexive thematic analysis. Quantitative data were analysed using descriptive and inferential statistics.

**Results:**

Four themes were identified which embody conditions that appear to be critical for working towards physical activity being embedded in local policy & practice within the Bradford District. These included: collaboration and sector integration, co-productive working, governance and leadership, and cultivating a learning culture. The process of co-producing a district-wide strategy for physical activity was key to facilitating shared ownership of the physical activity agenda across different levels of the system, and for supporting and maintaining cross-sectoral collaboration. On average, survey respondents connected with four more local organisations in relation to the physical activity agenda in January 2024 than in December 2021.

**Conclusion:**

Taking a partnership approach, and fostering a culture of evidence-informed decision making, is key to embedding physical activity into policy and practice at a local level. Investing time to understand the aims and values of each partner, and potential synergies and tensions between them, can support the development of a positive and productive collaboration and, subsequently, more effective whole-system delivery and population-level increases in physical activity.

**Supplementary Information:**

The online version contains supplementary material available at 10.1186/s12966-024-01704-5.

## Background

 The World Health Organization (WHO) describes physical inactivity as a pandemic, estimating that over 27.5% of adults and 80% of children do not meet physical activity guidelines, and trend data showing no improvement in inactivity prevalence over the past 15 years [1]. Physical inactivity can have substantial consequences for health and wellbeing, at both an individual and governmental level [2]. Physical inactivity contributes to over 20 chronic health conditions, costing the UK’s National Health Service around £1 billion per year [3, 4]. In addition, physical activity patterns differ according to socio-structural characteristics such as deprivation, ethnicity and gender, which contributes to widening health inequalities [5]. To address this challenge, over 90% of nations have developed a national physical activity policy [6]. National and regional policies are key enablers of population-level physical activity as they can increase opportunities to be active, help leverage funding, and support coordination of activities across sectors, organizations, places and stakeholders [7].

Policy change is integral to whole-system approaches that create social impact more effectively [8, 9]. To address physical inactivity, the interconnected nature of factors and actors that make up a political and social physical activity ecosystem must be understood [10]. A lack of social support for policy can restrict societal change; therefore, collaborative policy reform, that views policy change as a process involving stakeholder buy-in, is key to success [11]. Within England, the UK government has developed strategies and guidance that include co-ordinated efforts aligned with whole of systems approaches, as well as invested substantial funds to help implement them. For example, Sport England (a non departmental Government organisation) launched their Towards an Active Nation Strategy (2016) which included the funding of 12 Local Delivery Pilots (LDP) in 2018 to help tackle inequalities and make physical activity more accessible to more people. Sport England’s most recent strategy, Uniting the Movement (2021), focuses on funding specific investment programmes within local communities as part of a whole systems place-based approach to reduce physical inactivity and health inequalities and connect the places and spaces which make it easier for people to be active.

In the UK, local authorities have a role to play in local decision-making, the development and delivery of physical activity-relevant policy, and funding allocation. Multi-sectoral working is key to policy change, and evidence from the urban and transport planning sector suggests this may be more feasible at a local - compared to national - government level [12], provided they have incentives, resources, and capacity needed to make evidence-informed policy decisions. There is progress towards a devolved government structure in England, which transfers powers and funding from national to local government and ensures that approaches are tailored to the local culture and context [13]. Despite this, most physical activity policy research has focused on the national level and there is scarce evidence related to embedding physical activity in policy and practice at the local level [6, 7].

There are significant inequalities related to physical activity for Bradfordians and physical activity levels in Bradford are substantially lower than the national average [14, 15]. Situated in West Yorkshire, Bradford is the fifth largest local authority in England. Bradford is ethnically diverse and economically deprived with 14 of Bradford’s 30 wards^1^ being in the 10% most deprived in England. Since 2019, Bradford has received significant investment from Sport England as an LDP, to implement novel, systemic, joined-up approaches to tackling physical inactivity [16]. The current study presents a case study of a local approach to developing and embedding physical activity in policy and practice in Bradford, UK. This paper aims to generate evidence on the strategic development and embedding of physical activity in policy at a local level, by examining the factors contributing to systems change through a mixed-method case study.

## Study context

### Bradford Physical Activity policy and practice context

Physical activity system leaders across health and social care, public health, and sport and recreation are increasingly taking a joined-up approach to embedding physical activity in policy and practice in Bradford. This started with the establishment of Active Bradford (a consortia of member organisations) in 2015 as a recognition of the number of system partners that have a role to play in increasing physical activity. This was followed in 2017 by the whole systems approach to obesity (now called Living Well) and more recently by the addition of the JU: MP LDP.

A Bradford District cross-sectoral physical activity strategy group (herein described as strategy group) was established in 2020 as a mechanism to facilitate work linking up across the system in Bradford. Despite fluctuation in membership of this core group, it has included sustained senior-level representation from key organisations and programmes; see Table 1. Members attend the strategy group as part of their professional roles. The strategy group reports into the Districts’ Health and Wellbeing board, the lead partnership for the District.

**Table 1:** An overview of core membership of Bradford’s physical activity strategy group

Organisation / programme	Description
Active Bradford	Bradford District’s Sport and Physical Activity partnership, made up of member organisations who are committed to making it easy for people in Bradford to live an active, healthy life.
Active Travel (City of Bradford Metropolitan District Council)	Part of the Council’s Planning, Transport and Highways department, focused on developing the use of active travel within the community as a way to develop a healthier lifestyle.
Culture, Sport and Leisure (City of Bradford Metropolitan District Council)	Managing the Council’s Cultural and Sports assets to contribute to a better quality of life for all citizens of the Bradford district.
JU:MP (Born in Bradford, Bradford Teaching Hospitals NHS Foundation Trust)	Bradford’s whole-system approach to increasing physical activity and reducing inequalities in children and families in North Bradford, funded by Sport England. Born in Bradford, a research programme hosted by BTHFT, is leading the programme on behalf of Active Bradford.
Living Well (City of Bradford Metropolitan District Council, Bradford District and Craven health and Care Partnership)	Living Well is Bradford’s whole-systems’ approach to improving health and wellbeing for all across the district. The Living Well mission is to enable everyone to work together to make it easier for people in the district to adopt a healthier, more active lifestyle.
Yorkshire Sport Foundation	A National Lottery funded charity and Active Partnership working across the nine districts of South and West Yorkshire to create a vibrant, healthy and prosperous Yorkshire though everyone moving more.

The strategy group leads the physical activity agenda across the District. A key activity of the group is the co-production and delivery of a district-wide physical activity strategy, ‘Every Move Counts’. Strategy development was informed by a series of workshops with over 100 cross-sectoral stakeholders and senior leaders, and comprehensive consultation with over 1,100 Bradford residents. The strategy, which includes nine priorities for action (schools, children and young people, neighbourhoods and communities, sport and active recreation, health and social care, workplaces and workforce, greenspace, built environment, active travel, communications and campaigns) was launched in January 2024 [17]. The strategy group members advocate for change in policies and practices of wider district departments and aim to ‘connect the dots’ for maximum impact related to physical activity across their respective programmes and organisations.

### Research and evaluation context

This study forms part of a wider programme of research and evaluation connected to the JU: MP LDP programme (see Table 1). A key part of the Policy and Strategy theme within JU: MP focused on the work stream around strategic influence and ‘embedding physical activity in policy and strategy’ [15]. This work stream aligns with the pre-existing district partnership approach; it is a collaborative endeavour and therefore complements and forms part of other initiatives, such as Living Well (see Table 1). A process evaluation of JU: MP is being conducted, which aims to understand how JU: MP contributes to change within communities and across local policy and strategy systems [15]. The research presented herein is a ‘sub-study’ of the overarching JU: MP process evaluation. This research also contributes to a wider national evaluation of the Sport England-funded place-partnership work, the ‘National Evaluation and Learning Partnership’ (NELP), led by Sheffield Hallam University.

## Methods

A mixed-method case study design was employed to examine the impact of a partnership approach to embed physical activity in local policy & practice in Bradford, UK. A convergent parallel mixed-methods design was utilised, whereby data from qualitative and quantitative methods were collected simultaneously and analysed separately [18]. Quantitative methods were employed to examine whether change occurred, and qualitative methods were employed to unpick factors influencing change, and wider impacts. Given the explanatory focus of the case study, the qualitative component is the core of the study, and the quantitative component is supplementary [19]. Presentation of recruitment, data collection and analysis are aligned with a mixed-method reporting tool [20]; see additional file 1. The study received ethical approval from Leeds Beckett University in March 2020 (ref: 69870) and was transferred to and approved by University of Bradford in May 2021 (ref: E888). All participants consented to taking part in the study.

### Sampling and recruitment

A purposive sampling technique was employed for this study. Participants were selected based on their direct or indirect involvement in work to embed physical activity in policy and practice in Bradford. There were two main participant groups (1) strategy group members, and (2) wider stakeholders such as council officers, councillors, and community organisation representatives. Participants were invited to take part in all aspects of the study that they were eligible for based on their role; see Table 2. Recruitment was primarily facilitated by email. Participants completed a short demographic survey which also recorded their consent for participating in the study.

**Table 2:** An overview of data collection methods including participants and timings

Method	Objective	Participants	Timing
Meeting observations	Observation of physical activity strategic influencing meetings to document and reflect on factors influencing the work	Strategy group membership numbers have expanded as the district’s whole system approach has evolved, but it has ranged from 8 core members when it was first established to approximately 20. Indicative roles include the JUMP LDP Strategic Director, Active Partnership CEO, Bradford City Council Sport and Physical Activity Manager, Traffic and Road Safety Principal Engineer, Deputy Director of Public Health, Parks and Green Space Manager, Living Well Lead	Every other meeting (once every 2 months) from Jan 2021 - Jan 2024
	Observation of key (co-production) workshops with wider stakeholders to document and reflect on factors influencing the work	Workshop participants included those from a range of sectors (e.g. public health, environment, neighborhoods, children and young people, sport and recreation and communication and marketing) with an interest in the district’s partnership approach to supporting physical activity (such as council officers, councillors, and community organisation representatives). Participant numbers fluctuated but ranged from 70–90 participants attending each workshop	Six workshops, held in Dec 2021, May 2022, July 2022, Sept 2022, March 2023, and Jan 2024.
Semi-structured interviews and focus groups	Interviews and focus groups with the strategy group to understand barriers and facilitators, and change mechanisms related to embedding physical activity in policy and practice	Strategy group membership numbers have expanded as the district’s whole system approach has evolved, but it has ranged from 8 core members when it was first established to approximately 20. Indicative roles include the JUMP LDP Strategic Director, Active Partnership CEO, Bradford City Council Sport and Physical Activity Manager, Traffic and Road Safety Principal Engineer, Deputy Director of Public Health, Parks and Green Space Manager, Living Well Lead	Six interviews (Aug 2021), one focus group (5 participants) and two interviews (Aug 2022), two interviews and one focus group (7 participants) (Aug 2023)
	Focus groups to understand experiences and impact of co-production activities	Sub-sample of wider stakeholders (such as council officers, councillors, and community organisation representatives)	Two focus groups in June 2022 (13 participants)
Policy and feedback surveys	To characterise the sample and understand barriers and facilitators, and change mechanisms related to embedding physical activity in policy and practice	Strategy group & wider stakeholders (such as council officers, councillors, and community organisation representatives)	Surveys in Dec 2021 and Jan 2024, and a Dec 2021 workshop follow-up survey.

### Data collection methods

Data collection occurred at multiple timepoints between January 2021 and January 2024, and involved a range of methods including interviews and focus groups, observations of key co-production activities, and surveys; see Table 2.

### Qualitative methods: observations, interviews and focus groups

Through immersion in the setting, qualitative observations permit a deep understanding of context, behaviours, and interactions [21]. Regular observations of strategy group meetings, and key co-production workshops, were guided by an observational summary sheet, which focused on capturing activities and interactions, informed by Spradley [22]. Observations were supplemented by semi-structured interviews and focus groups with key informants, which permitted in-depth reflection on embedding physical activity in policy and strategy from the perspective of those leading the agenda in Bradford (see additional file 2 for the topic guide). Focus groups and interviews were audio recorded and transcribed verbatim.

A feedback survey was administered to allow participants to share their experiences and perceived impact of the co-production workshop. These experiences were explored in more depth in online focus groups with a sub-sample of workshop attendees.

### Quantitative methods: policy survey

The policy survey consisted of four sections: sample characteristics, an adapted Theoretical Domains Framework (TDF), physical activity policy action, and social network analysis. Self-reported sample characteristics included: age, gender, ethnicity, highest educational level, employment (sector, organisation, role), and involvement in developing and/or implementing local policy and strategy. The adapted version of a valid TDF [23], explored behavioural barriers and enablers to embedding physical activity in policy and practice amongst stakeholders. The physical activity policy action was novel to this study and focused on the appropriateness, embeddedness, and operationalisation of physical activity. The social network analysis was of a bespoke design to understand connectivity and collaboration across the system [24]. It captured the frequency in which stakeholders connected with a range of local organisations related to physical activity. The survey was administered electronically (via email) using SmartSurvey (https://www.smartsurvey.co.uk*);* or in person during a workshop.

### Data analysis and integration

Qualitative data was analysed using inductive, reflexive thematic analysis [25]. Two researchers (JH and EL) independently and inductively open coded all data, before independently crafting themes from across all data sources. Three researchers (JH, EL and AC) had three meetings to review the two initial sets of themes and develop the themes as presented. A ‘critical friend’ approach to the discussion encouraged reflexivity and helped maintain rigour [26].

Analysis of the survey data was carried out in Stata version 17.0 (StataCorp LLC, TX, USA). Continuous participant characteristics were calculated as mean (standard deviation [SD]) and categorical characteristics as N (%). Paired t-tests were used to analyse timepoint (Baseline and 24-months) differences for: number of local connections; policy implementation; and policy embedding. Ordered logistic regressions were used to assess the association of categorical survey answers. The logistic models were restricted by the nature of the potential covariates that were collected during the study, as such all models were unadjusted and set with a single independent/dependent variable comparison. The models assessed: the perceptions of capability, opportunity, and motivation to include physical activity in practice; and physical activity policy implementation; differed depending on employment sector (Health, Sport & active recreation, Education, Environment, Neighbourhoods, Other). The constant in each model was set to those working in a ‘Health and Social Care Sector’. Statistical significance for all analysis was set at the alpha level of 0.05. To translate the outcomes of each model, prediction equations were used to establish the likelihood (percentage chance) of participants from each employment sector scoring the highest response to questions. Outputs from all models are presented in Additional file 3.

The network analysis data was acquired through likert scale questions contained in the survey, about the extent to which participants connect with organisations, and how much these are about physical activity. Answering the survey with a response on point 2 of the likert scale to both questions created a connection between the answering participant and the organisation. This data was then processed in R (version 4.2.0) [27] and visualised as a network map using the igraph package (version 2.0.3). A line on the network map represents a connection between two organisations, the thickness of the lines is proportionate to the amount of people within an organisation who had responded as connecting with the organisation that line connects to.

Integration of qualitative and quantitative findings enriches knowledge and fosters transformative change [28]. Four researchers (JH, EL, AC and ND) engaged in collective sensemaking to produce an ‘integration through narrative’ [29] i.e., thematic alignment of the qualitative and quantitative data, driven by the qualitative findings.

## Results

135 individual stakeholders participated in this study; 26 took part in between one and four interviews or focus groups. Policy surveys were completed by 66 stakeholders in December 2021 (40.2% response) and 84 stakeholders in January 2024 (44.1% response). Descriptive characteristics for those who completed the participant survey are presented in Table 3. Time-point analysis indicated a significant increase in the number of connections made by participants (*p* = 0.02), but no change in perceptions of the embeddedness of physical activity within policy (*p* = 0.993) or practice (*p* = 0.304) (Table 4). When compared to the Health and Social Care sector, those working in the sport and active recreation sector had higher ranking survey outcomes across all questions relating to; capacity, opportunity, motivation, and implementation (Additional file 3, Tables S1 &S2).

**Table 3:** Descriptive characteristics

Characteristic	Value
Age (years)	47.32 (9.0)
Sex, n women	66 [52.0]
Ethnicity, n White	98 [77.2]
Employment Length, n 10 + years	77 [60.6]
Qualification status, n bachelors or higher	100 [78.7]
*Employment sector*	
Health	19 [15.3]
Sport & active recreation	43 [34.7]
Education	12 [9.7]
Environment	27 [21.8]
Neighbourhoods	9 [7.3]
Other	14 [11.3]

Values presented as Mean (SD) or n [%]

**Table 4:** Difference in outcomes between baseline and 24 months

Variable	Mean Difference	95% CI	*p*
Total local connections	**3.48**	0.57, 6.40	0.020
Embedding policy	**0.23**	−0.49, 0.49	0.994
Implementing policy	**−0.01**	−0.21, 0.67	0.304

Bold = significant at alpha 0.05

Four themes were crafted from the qualitative data which embody conditions underpinning the embeddedness of physical activity in local policy and practice. These include (1) collaboration and sector integration, (2) co-productive working, (3) governance and leadership, and (4) cultivating a learning culture. Whilst the themes are presented separately they are interconnected, for example developing co-productive working practices (theme two) supported both collaboration across organisations (theme one) and cultivating a learning culture (theme four). Within each theme, attention is paid to what worked, how, and the enabling and constraining influence of contextual factors, for facilitating change related to these conditions within Bradford.

### Theme one: collaboration and sector integration

Stakeholder narratives indicated that collaborative working, within and beyond the physical activity strategy group, is required to effectively and consistently embed physical activity in local policy and practice. This theme demonstrates the importance of negotiating complex challenges to maximise the benefit of collaborative and cross-sector leadership of the physical activity agenda locally.

Across the district there were many individuals and organisations whose remit was focused on or included physical activity promotion, however the leadership and capacity required to unify as a collective was initially lacking. As one participant explained, investment from Sport England provided leadership capacity and energy to develop collaborative working practices between and across these key individuals and organisations:

*“There’s no point having £10 million and then saying you don’t have the resources to do strategic influence for physical activity across the city*,* we do have resource*,* we just have to shift it… getting the key leadership players across the city aligned and clear and supportive”* (Interview).

Once the physical activity strategy group was established, an initial challenge to partnership working was the collaborative inertia in communicating and understanding the different strategy group members’ perspectives and the aims and visions of their respective organisations and programmes. For example, the framing of physical activity differed by employment sector with sport and recreation sector stakeholders tending to adopt a holistic view of physical activity, whereas for others, physical activity was one component of a wider (health) agenda. This made decision-making complex and time-consuming:

*“There’s a risk of physical activity being put into an obesity box if it is being presented alongside food. And physical activity becomes only about obesity… There’s also the economic and the inclusive growth and the skills agenda*,* and the community development agenda… We just need to be mindful and let’s keep the broad width of physical activity”* (Interview, sport sector).

However, through developing a shared understanding of key partner organisations, and recognising synergies and tensions between different agendas, the strategy group established collective aims, trust and collaborative working practices. An initial investment of time was needed to build trust and establish positive collaborative working practices, which helped to eliminate organisational silos:

*“I think what we’ve struggled with before is [organisation] is very much*,* owned by [sectors] and we actually want it to be owned across the system and I think we’ve done a lot of work with [organisation] to kind of work*,* more towards that way of working*,* so it’s not about us delivering things or claiming the glory*,* it’s about those coordinated actions”* (Interview).

One clear area where there was reciprocal benefit to collaborative working was in relation to funding. Partnerships were able to overcome financial instability within the system by pooling resources to create opportunities for physical activity across Bradford:

*“Also comment re linking place-partnership work in with ‘play zones’ to maximise on funding and matching capital and revenue investment for maximum impact*,* i.e. need to think about how we ‘harness’ existing work…. discussion about regeneration (Capital) work that is planned for the district – thinking about how we can ‘draw on’ other investments to make best use of finances we do have*,* as the Local Authority do not have any revenue to spend on these capital investments”* (Observation data).

Physical activity strategy group stakeholders agreed that taking a multi-pronged approach to collaborating with wider system stakeholders, involving ‘depth’ and ‘breadth’ work, was required to embed physical activity in local policy and strategy. Stakeholders recognised the value of in-depth collaborative working with specific stakeholders and teams, around specific work areas, to embed physical activity into wider policies. For example, one of the areas where this was particularly successful was embedding physical activity within Bradford’s children and young people plan as it was perceived to enable progress to happen more quickly and have greater potential to impact on those most in need. This was reported to be facilitated by the inclusion and authentic involvement of a range of stakeholders, and by allowing the time needed to build relationships with partners via repeated engagement in the right meetings (working groups) with the right people:

*“It’s been a really big win… now it’s [physical activity] in the [children and young people’s] plan*,* really*,* as an enabler of wider health and social outcomes… I was just really working to meet with the key decision makers and build relationships and go to the important meetings and just advocate and influence*,* raise the profile*,* those big workshops*,* bringing everyone together definitely helped*,* but also just making sure I was in*,* we were in on the working groups”* (Interview).

Cross-sector collaborative working was also seen as fundamental to the successful development and operationalisation of a district-level physical activity policy (breadth); see theme two.

### Theme two: co-productive working

This theme highlights how co-productive working was key to galvanising stakeholders across the local system around physical activity. It primarily draws on data related to the co-production processes which occurred as part of developing the Bradford District physical activity strategy.

Stakeholder narratives indicate that the co-production process facilitated ‘buy in’ of stakeholders systemwide. Attending co-production workshops enabled stakeholders to gain insight into and an understanding of the benefits of physical activity, including how supporting physical activity initiatives can align with and contribute to their professional priorities. This contributed to stakeholders placing more value on physical activity, and the development of shared ownership and responsibility across the system to maximise opportunities for physical activity:

*“We have got more partners around the table*,* that now understand physical activity and are more engaged and want to work with us”* (Interview).

**Fig. 1:**
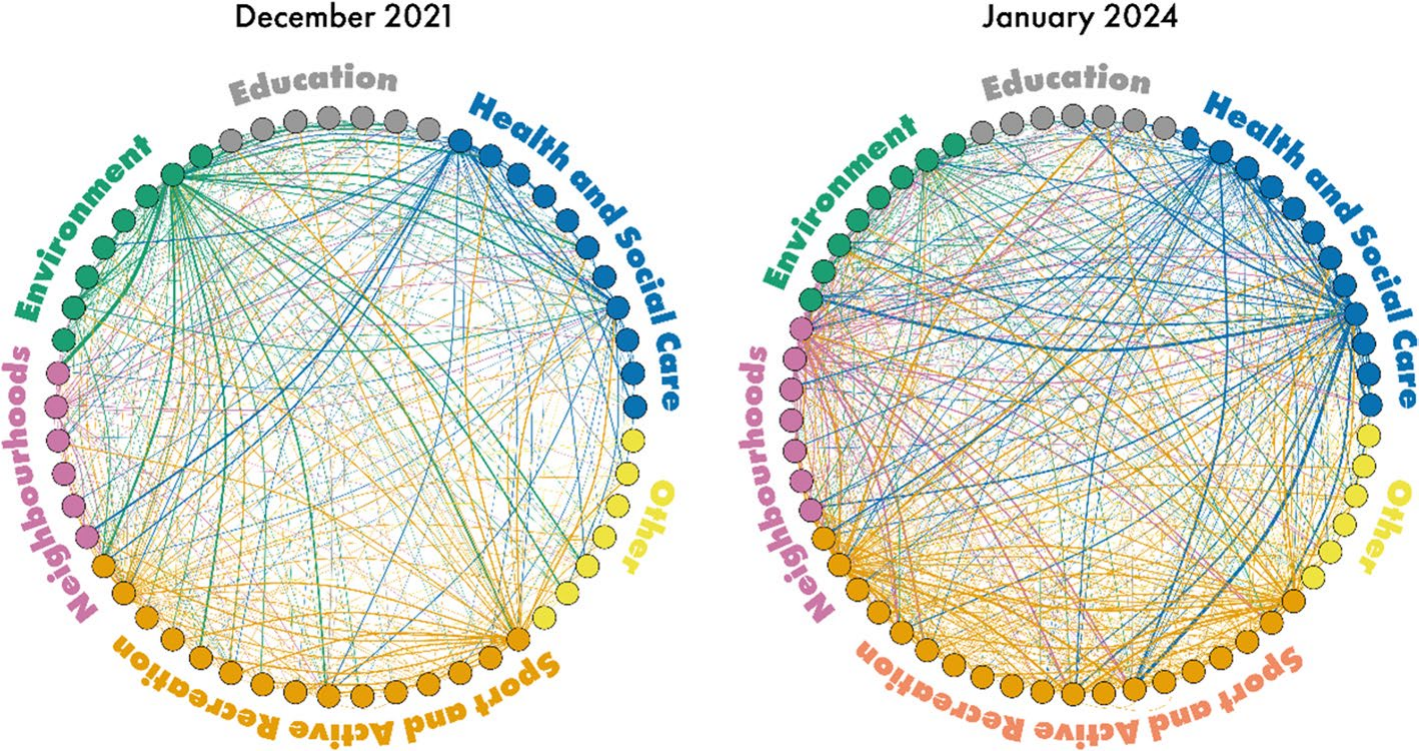
Network map showing all connections between organisations reported in December 2021 and January 2024. Each node represents an organisation. Each vertex represents a connection to another organisation. The width of the vertex represents the weight of connection, for example where multiple individuals reported connecting from and to the same organisation. Organisations have been grouped and coloured according to their sector. The colour of the vertex indicates the parent sector of the person reporting the connection. Organisations are organised alphabetically (anticlockwise) within each sector

The inclusion of a wide range of stakeholders in the decision-making process, for example from health and social care, sport and recreation, neighbourhoods, and the built environment sectors, fostered greater collaboration, within and across sectors, through the sharing of ideas, skills and experience. The number of connections made by participants with other stakeholders across the system significantly increased throughout the development process (~ 3.5 per person, *p* = 0.02). Figure 1 (network map) illustrates a greater density of connections between surveyed organisations in 2024 than in 2021. In 2021, most cross-sector collaboration was driven by the Environment and Sport and Recreation sectors whereas in 2024, all but the Education sector had a greater number of connections with other sectors compared to 2021.

64% of open-text responses to a workshop follow-up survey focused on the value of the co-production workshops in supporting networking opportunities and/or connecting with others. The opportunity for cross-sector discussions beyond stakeholders’ ‘usual networks’ provided through the process of co-producing the physical activity strategy encouraged collaboration across sectors and organisations:

*“We were in slightly more diverse groups and that was interesting in bringing together [people] that I wouldn’t normally talk to and as [name] said on this call*,* it helped to build those links or at least demonstrate the breadth of knowledge and the feeling in the room about these topics… I think if I was pinpointing something it would definitely be that”* (Focus group).

However, the process of developing the strategy took just over two years and some stakeholders were concerned that it represented “all talk and no action”. That is, that the time-consuming nature of co-productive working may have negatively impacted progress towards implementing and embedding the physical activity in policy, suggesting that there may be an ‘opportunity cost’ associated with investing significant resource into the co-production process:

*“If we’d have put some of that energy into* *doing[emphasis added]… you know*, *even a third of the time spent writing the strategy…. it would have been transformative”* (Focus group).

### Theme three: governance and leadership

This theme highlights the importance, and complexities, of establishing both appropriate governance structures and senior leadership support, to maximise the effectiveness of efforts to embed physical activity in policy and practice. Participants highlighted the importance of effective decision-making and accountability systems. It took time, and knowledge of the existing system context, to establish acceptable overarching governance structures. One participant shared how this was difficult to achieve within Bradford due to the multiple actors and organisations within the system:

*“The other added complexity really is a kind of broader partnership for physical activity. We’ve got the work of [department]*,* we’ve got the work of [organisation]… And then we’ve got [organisation]… If we were to step back and say*,* ‘right*,* this is the approach and this is the governance and this is accountability. What does that actually look like?’ That we haven’t quite got to yet. As I say it is a work in progress because we are*,* things are progressing*,* and certainly the relationships within that are really good relationships. It’s just about getting clear on roles and responsibilities”* (Interview).

There was a view that strategic leadership of physical activity across the District should involve - but be independent from - the local authority, due to a perception amongst communities that council leaders and employees may act in a way that is biased towards a local political agenda:

*“It’s very important for Active Bradford to be independent from the local authority and sit within its own right*,* in terms of governance and leadership. And there are lots of benefits around that. What we don’t want is the carrot and stick from the local authority. So we’ve worked extremely hard to ensure that Active Bradford is independent to local authority*,* has its own voice and influence and can negotiate and meander around the decisions that it needs to make… the reason for that was previously when we had the community sports network it was very much seen that that was driven by the local authority and the ambitions of the local authority. That’s not to say that Active Bradford doesn’t sit within the ambitions of the district plan for Bradford. Of course it does*,* but it holds its independence”* (Interview).

Distributed leadership through the establishment of thematic leads and action plans for different themes (such as green space, children and young people) that report into the strategy group, provided clear lines of responsibility and accountability for different components of the work. These factors were viewed as critical for driving change in practice, through increasing understanding of the objectives, role clarity, and ownership across stakeholders at all system levels:

*“There is a launch event planned… where we want to bring all of the thematic leads and advocates of the Physical Activity Strategy on board to help embed the work… using their various expertise in the field of physical activity to put the Action Plan into action*” (Focus group).

Alongside distributed leadership across the system, stakeholders also referred to and provided examples of the importance of top-down approaches to leadership, including the backing of District senior leaders at the highest level:

*“So key people like [BIHR director]*,* like [CEO of council]*,* like [leader of the council] so this is leadership right from the top… because if they don’t have confidence in something*,* it’s not going to go anywhere”* (Interview).

Such individuals, such as the CEO of the Council, were able to exert influence by ‘opening doors’, leveraging support and increasing the visibility of the physical activity agenda in high-level environments across the district.

*“It was evident that the CEO of the council has the power/position to ‘make things happen’ when it is something she is on board with*,* and she was certainly behind the physical activity strategy and related aims at the meeting today”* (Observation notes).

Participants expressed that senior leaders were more likely to exercise their influence positively when they had ‘confidence’ in the delivery team and their approach to embedding physical activity in policy and practice. In particular, stakeholders felt that the co-production process was key to developing confidence and trust amongst senior leaders, as it provided visibility and transparency across multiple levels of the system whereby individuals felt empowered through the community driven approach which also gave credibility to the process:

*“That community engagement… it’s helped to galvanise our partners… and that for me is also why relationships [with senior District leaders] have improved… ‘Oh*,* actually*,* it’s not just public health telling you this problem. We’ve actually got communities telling us this and we have over 1000 people that contributed towards this.’ And that also was a fundamental part of why I think things have developed and improved [with senior leadership]*” (Interview).

### Theme four: cultivating a learning culture

This theme was developed to describe how positive change was facilitated by embedding learning as an integral part of the policy and strategy process. A strong learning culture within the physical activity partnership work was perceived as instrumental to achieving significant impact. This was valued and supported by physical activity strategy group stakeholders. A key facet was the embedded researchers who were working alongside system partners and legitimised by the purpose of conducting the process evaluation.

The mutually beneficial relationship provided a bridge between academic knowledge and critical approaches to developing processes and ways of working which contributed to the common goal of embedding the physical activity policy and strategy across the District. Critically, the researchers supported reflective practice through methods such as Ripple Effects Mapping (REM) and focus groups. This enabled context-specific capacity building whereby the strategy group considered what worked (and did not work), to inform an iterative approach to embedding physical activity in policy and practice.

Working *with* system partners in this way rather than *on* or *for* them, provided a sense of objectivity to evaluate which allowed feedback mechanisms to be integrated into the delivery process. For example, reflecting on the ‘failures’ of previous strategic working around physical activity – through focus group discussion - was instrumental to developing components of the current approach, including designating ‘thematic leads’ and creating clear, tangible action plans:

*“The problem with the old strategy was… there was no ownership of the actions. There were plenty of actions. There were good actions. They could’ve happened… there was no responsibility for who was leading on… so whether it’s physical activity to health pathways for example*,* or whether it’s getting older people active*,* you know*,* who’s really doing that*,* you know*,* no responsibility at the time… That’s the change I want this time”* (Focus group).

The wider partnership valued the research input which drove a systematic, evidence-informed approach to physical activity in local policy and practice. For example, a ‘policy mapping’ activity that involved assessing the extent to which physical activity was embedded into existing local policies provided direction and energy after a period of relative stagnation, due to the enormity of the task, and differing opinions regarding how to approach embedding physical activity in policy. This research-led activity allowed stakeholders to develop a shared understanding of key opportunity areas and a shared vision:

*“That meeting was really good*,* like all the attendees really enjoyed it [policy mapping]*,* they were like*,* ‘oh this is amazing work that we’re doing’… if it’s engaging these really quite senior people*,* if it’s engaging them in this work*,* you know*,* that is a positive outcome”* (Interview).

## Discussion

This mixed-methods case study examined the factors underpinning physical activity systems change at a local government level (Bradford, UK). We found that a combination of ‘breadth’ and ‘depth’ work, involving consistent advocacy efforts, is required to embed physical activity in policy and practice. We identified changes in conditions underpinning the embeddedness of physical activity in policy and practice. These included: (1) increased collaboration across organisations and cross-sectoral working, (2) establishing consistent co-productive working practices, (3) developing appropriate governance structures and leadership processes, and (4) cultivating a learning culture. Despite local policy approaches being key to increasing population-level physical activity, research to date has focused at a national-level [6, 7]. This study provides original insights that will support the development and delivery of effective sub-national approaches to physical activity promotion.

Overall, the findings of the present study align with existing and emerging frameworks for place-based, whole-system and policy approaches to public health. A recent systematic review of whole-system approaches to obesity identified several features of successful approaches which included full engagement of relevant partners and the community, local evaluation, and good governance [30]. The National Evaluation and Learning Partner (NELP) for the Sport England Local Delivery Pilots have developed a conceptual framework that outlines 10 conditions for place-based working with respect to physical activity. Six of these conditions overlap with the findings of the present study: distributed and collective leadership, community power (local people-led initiatives), cycles of learning and action, collaboration across organisations, workforce capacity and capability, and processes for agile, collaborative working [31]. This discrepancy in that four don’t align (understanding barriers and enablers, focus on inequality and intersectionality, cultural and social norms, physical environments), is likely due to the focus in the present study on the process of policy development and strategic working, rather than the enactment on policy which will likely address the four remaining conditions. A focus on policy and strategy represents one type of whole-system approach and/or one component of a broader whole-system approach to physical activity [32]. The conceptual framework was developed based on learning from multiple places that adopted technocratic policy, asset-based community development, and/or tackling structural inequality approaches [32]. Thus, the present study adds to the empirical evidence-base in support of the conceptual framework for place-based working around physical activity.

We found no difference in perception of embeddedness of physical activity in policy over time, which is unsurprising as policy change takes time to embed, and most Bradford District policies were not reviewed/updated during the study. Additionally, this may be reflected in the qualitative data which revealed several challenges to this partnership way of working. Survey data suggested that health and social care sector stakeholders were less likely to consider physical activity as well embedded within the policies associated with their role, than sport and recreation sector stakeholders (Additional file 3, Figure S1). This local picture mirrors the regional and national picture in Australia; a comprehensive policy audit revealed that primary and secondary healthcare (alongside workplaces and education) were the least frequently addressed domains across physical activity relevant policy action [8]. The qualitative data in the present study help uncover potential explanations for this phenomenon; for health sector stakeholders, physical activity was one component of a wider health agenda and may not always be relevant (for example, to smoking cessation). Whereas for the sport and recreation sector, physical activity can be embedded within almost all priorities.

The findings highlight the importance of working collaboratively within and across sectors to maximise the embeddedness of physical activity within local policy and practice. Leadership capacity appears to be key to bringing together key stakeholders and galvanising partnership working; in Bradford, this was provided through the Sport England-funded Local Delivery Pilot and the councils public health programme. It is likely that the nature of both of these programmes being underpinned by a whole systems approach, facilitated the establishment and maintenance of the physical activity strategy group. Connections between different stakeholders and organisations related to physical activity were more prevalent when the Physical Activity Strategy was launched in early 2024, than at the start of the process in 2021. This suggests that the partnership approach to embedding physical activity in policy and practice in Bradford has contributed to increased collaboration.

Another key finding within the current study was the perceived importance of establishing appropriate governance and leadership structures for enabling the integration of physical activity in policy and practice, despite the challenges of doing so when taking a whole system approach involving various organisations and stakeholders. A similar finding was reported by Beaudart and colleagues [33], which evaluated Liverpool’s development of the 2005–2010 physical activity agenda and identified the value of robust partnership structures to steer project development. The Liverpool Sports and Physical Activity Alliance was established to oversee and improve the synergy across different elements of strategy implementation, which facilitated the integration of physical activity into other strategies [33]. The importance of ‘good governance’ is also emphasised in a recently published guide, Getting Australia Active III, which aims to build greater understanding and capacity among government policy makers to employ a whole-of-systems approach to increase physical activity in Australia [34]. The authors define ‘good governance’ as “decision making, policy creation and rule enforcement that is non-discriminatory, participatory, has integrity, is transparent, efficient (not wasteful) and is subject to accountability (if someone does the wrong thing)” [40]. Intersectoral governance arrangements can avoid narrow formation and imbalanced framing of policy [35, 36], and support collaboration when implementing policy [37]. Within the present study, the development of ‘thematic leads’ for priority areas (such as green space) was viewed as a positive step towards distributed leadership and intersectoral governance, as responsibility is shared and dispersed amongst stakeholders in different organisations and teams [38]. Such an approach reflects an ‘enabling leadership’ style that draws on both adaptive and administrative leadership practices to facilitate the stimulation of new ideas and collaborations, embedding these into a formal, coordinated system [39].

Using a co-productive approach to developing Bradford Districts Physical Activity Strategy, by involving stakeholders from different sectors and levels of the system and extensive community consultation, was found to be an effective approach to achieving ‘buy in’ to the physical activity agenda and fostering ownership amongst stakeholders. The importance of engaging partners from the outset to maximise widespread ownership of a physical activity strategy and agenda has also been reported elsewhere [33]. There is a growing evidence-base, outside of the physical activity strategy arena, showing the positive impacts of co-producing interventions on stakeholder engagement [40–42]. We found that a co-production approach was particularly important for engaging senior leaders, as it increased their confidence that the work is community-driven. Senior leadership buy-in is critical to system change to allocate appropriate resources and encourage middle managers to prioritise implementation [33, 43]. Our findings, alongside wider literature, challenge an assumption that the ‘content’ of policies is their most important attribute [44] and highlight the importance of the *process* of policy development for embedding physical activity in practice. Indeed, co-productive working practices may reduce the policy-to-practice disconnect [44] and it is important that co-production continues throughout the implementation and learning cycles of the policy process. The present study also highlighted a potential ‘trade off’ of co-productive working; genuine co-production is time-consuming and resource intensive [45] and the ‘opportunity cost’ should be carefully considered.

Cultivating a learning culture was found to support the embedding of physical activity in policy in practice in Bradford. Engaging in REM workshops enabled stakeholders to iteratively embed learning into practice to contribute to continuous improvement. REM is a visual, participatory method to capture wider intended and unintended impacts of initiatives [46]. A recent methodology paper provides evidence that REM can be engaging for practitioner stakeholders and reduce the research-practice gap [47]. In the present study, the lead researcher was a member of the physical activity strategy group and contributed to delivery which, in practice, meant that there were less rigid boundaries between implementation and evaluation than traditional research projects. This approach aligns with the ‘embedded researcher’ model in which researchers are integrated in practice settings. In line with extant literature e.g. [48, 49] we reflect that this approach permitted effective knowledge exchange and contributed to enhanced research capacity within the strategy group.

### Strengths and limitations

The methodological approach taken is a key strength of this work. Conducting embedded research over a prolonged period permitted a deep understanding of the factors underpinning the embeddedness of physical activity in policy and practice in a local context. Integrating qualitative and quantitative methods contributed to knowledge production beyond what would have been achieved through presenting and interpreting the results separately [28]. A limitation of the quantitative analysis was the small sample size, although this is not unexpected given the local nature of the policy implementation being evaluated. The cross-sectional nature of the analyses does limit the ability to assess causation, however insight is provided by the qualitative data. This study focused on evaluating the process of embedding physical activity in policy and practice at a local level. It is also imperative to evaluate the implementation and impact of policies to advance knowledge on what works, and how, in different contexts [13]. In Bradford, researchers are working alongside the physical activity strategy group to design and deliver a contextually relevant evaluation of Bradford District’s Physical Activity strategy; Every Move Counts, drawing on the Physical Activity Environment Policy Index [6]. We urge other localities to monitor and evaluate the implementation and impact of their local policies.

### Recommendations for practice

The findings from this study suggest several practical recommendations for maximising local approaches to embedding physical activity in policy and practice.

1. Collaboration and sector integration: It is important to establish a ‘working group’ comprising cross-sectoral system leaders with sufficient capacity within their roles to drive the work forward. To support the development of a positive and productive collaborative relationship, take time at the outset to understand the aims and values of each participating stakeholder, and consider synergies as well as potential tensions and how to manage them.
2. Co-productive working: Taking a co-productive approach to developing policy and practice is essential to developing shared responsibility and ownership across all levels and dimensions of the system. It is important to allow time to do this authentically which involves sustained interaction, facilitating opportunities for networking, and ensuring that stakeholders’ views are incorporated into developed policies. It may be beneficial to engage in implementation alongside co-designing policies and strategies to minimise any ‘resistance’ to co-production linked to a perception of ‘all talk, no action’.
3. Governance and leadership: Take time to establish appropriate governance structures and secure the support of District senior leaders at the highest level, as they are essential to ‘opening doors’ to truly embed physical activity across all levels of the local system. To do this, it is important to demonstrate to senior leaders that they can trust in your approach. Engaging in co-production with communities and cross-sectoral stakeholders is a useful strategy to do this, as it highlights widespread support and buy-in around the physical activity agenda.
4. Cultivating a learning culture: Engaging in formative evaluation and reflective practice can enhance local partnership approaches to physical activity. In the UK, there is a national strategy to boost research capacity and capability within local government, which is being operationalised through Health Determinants Research Collaborations (HDRCs). Partnering with experienced research professionals, through for example, local HDRCs, can help to embed a culture of using evidence to inform decision-making as part of approaches to embedding physical activity in policy and practice locally.

## Conclusion

This mixed methods case study research examined the factors contributing to systems change within a partnership approach to embedding physical activity in policy and practice in Bradford, UK. Through analysing interview, focus group, observational and survey data collected over a three-year period, we propose various conditions that underpin successful, strategic approaches to physical activity at the local level. Good governance and distributed leadership processes supported effective cross-sectoral integration and collaboration, co-productive working-built ownership across different sectors and levels of the system, and the cultivation of a learning culture supported increased evidence-informed and reflective practices, all of which served to strengthen the strategic approach to physical activity across the district. It is essential to evaluate local approaches to embedding physical activity in policy and practice across the UK and internationally, and this study provides a blueprint that can guide these efforts, to enable more effective, whole-system delivery and population-level increases in physical activity.

## Supplementary Information

Additional file 1. Mixed method reporting framework.

Additional file 2. Focus group topic guide.

Additional file 3. Analysis outputs.

## Data Availability

The dataset that generated and analysed during the current study are available from the corresponding author on reasonable request.

## Abbreviations

LDP: Local Delivery Pilot
NELP: National Evaluation and Learning Partner
TDF: Theoretical Domains Framework
WHO: World Health Organization

## Footnotes

1 Electoral districts at sub-national level represented by one or more councillors.
