# Supplementary material for: Taking a partnership approach to embed physical activity in local policy and practice: a Bradford District case study
Source: Int J Behav Nutr Phys Act. 2025 Jan 7;22:3. doi: 10.1186/s12966-024-01704-5 (PMC11707940; doi:10.1186/s12966-024-01704-5)
Supplement: Supplementary file 2 — Additional file 2. Focus group topic guide. [file 12966_2024_1704_MOESM2_ESM.docx]

Additional file 2. JU:MP policy and strategy focus group topic guide example

- What are your roles within the work to embed PA within policy and strategy? Have these changed over the last 6 months, if so, why and in what way?
- What have been your priorities over the last 12 months and why? Main success / challenges?
- In relation to your priorities over the last 12 months… What change were you hoping to see? (How) is the activity contributing to change? How do we know? Is change happening in some circumstances and not others? Are things changing in expected/unexpected ways?
- What about the intervention or the wider context has restricted or facilitated change? Or made it easier or harder to deliver the work?
- How is the strategic PA work being evaluated and why? How is this going? What helps/blocks this? Any challenges / benefits / impacts / learning?
- Who is represented within the strategic PA work? Whose voices are included/excluded and why and at what level e.g. meaningfulness of contribution? Challenges / impacts / learnings related to this?
- What does sustainability look like for this work? Are we working towards this now? Challenges / successes? What do we need to think about over the next 12 months?
- What processes are there to link this work into wider projects/systems (e.g. JU:MP, Living Well)? Are there any synergies/tensions? Challenges / benefits? How might this work better?
- Any other factors that have made it easier or harder to deliver the work… reflections / learning / things would do differently / priorities for next 6 months?
- Is there anything you would like to say about this work or JU:MP more broadly, or anything else you would like to add?
