# Supplementary material for: Taking a partnership approach to embed physical activity in local policy and practice: a Bradford District case study
Source: Int J Behav Nutr Phys Act. 2025 Jan 7;22:3. doi: 10.1186/s12966-024-01704-5 (PMC11707940; doi:10.1186/s12966-024-01704-5)
Supplement: Supplementary file 3 — Additional file 3. Analysis outputs. [file 12966_2024_1704_MOESM3_ESM.docx]

**Additional file 3 – Analysis outputs**

**Table S1.** Results of ordered logistic regression models for policy engagement questions by employment sector.

| **Employment Sector** | **β coefficient** | **Odds ratio** | **95% CI** | ***p*** |
| --- | --- | --- | --- | --- |
| *Parent & child influence* | | | | |
| **Sport & active recreation** | **1.254** | **3.504** | **0.286,2.222** | **0.011** |
| Education | 0.380 | 1.462 | -0.835,1.595 | 0.540 |
| Environment | 0.292 | 1.339 | -0.770, 1.354 | 0.590 |
| Neighbourhoods | 0.639 | 1.894 | -0.614, 1.891 | 0.318 |
| Other | 0.755 | 2.128 | -0.855, 2.366 | 0.358 |
| *Appropriate* | | | | |
| **Sport & active recreation** | **0.974** | **2.650** | **0.010, 1.939** | **0.048** |
| Education | 0.761 | 2.141 | -0.507, 2.029 | 0.239 |
| **Environment** | **1.889** | **6.614** | **0.699, 3.079** | **0.002** |
| Neighbourhoods | 1.362 | 3.905 | -0.013, 2.738 | 0.052 |
| Other | -0.079 | 0.924 | -1.335, 1.177 | 0.902 |
| *Embedded* | | | | |
| **Sport & active recreation** | **2.241** | **9.406** | **1.196, 3.287** | **0.000** |
| Education | 0.529 | 1.697 | -0.776, 1.834 | 0.427 |
| **Environment** | **1.155** | **3.175** | **0.103, 2.208** | **0.031** |
| Neighbourhoods | 1.346 | 3.843 | -0.107, 2.799 | 0.069 |
| Other | 0.939 | 2.557 | -0.519, 2.396 | 0.207 |
| *Opperationalised* | | | | |
| **Sport & active recreation** | **2.107** | **8.224** | **1.045, 3.169** | **0.000** |
| **Education** | **1.429** | **4.174** | **0.019, 2.839** | **0.047** |
| Environment | 1.130 | 3.096 | -0.001, 2.262 | 0.050 |
| Neighbourhoods | 1.194 | 3.301 | -0.238, 2.626 | 0.102 |
| Other | 0.200 | 1.222 | -1.169, 1.569 | 0.774 |
| Health sector employment set as reference; Bold = significant at alpha 0.05. | | | | |

**Table S2.** Results of ordered logistic regression models for the COM-B questions by employment sector.

| Employment Sector | β coefficient | Odds ratio | 95% CI | *p* |
| --- | --- | --- | --- | --- |
| *Psychological capacity* | | | | |
| **Sport & active recreation** | **1.346** | **3.841** | **0.432, 2.259** | **0.004** |
| **Education** | **1.333** | **3.792** | **0.116, 2.549** | **0.032** |
| **Environment** | **1.265** | **3.541** | **0.299, 2.229** | **0.010** |
| Neighbourhoods | 1.080 | 2.946 | -0.22, 2.386 | 0.105 |
| Other | -0.307 | 0.736 | -1.47, 0.865 | 0.608 |
| *Physical capacity* | | | | |
| **Sport & active recreation** | **1.610** | **5.000** | **0.663, 2.555** | **0.001** |
| Education | 1.056 | 2.875 | -0.23, 2.346 | 0.109 |
| **Environment** | **1.426** | **4.163** | **0.429, 2.423** | **0.005** |
| **Neighbourhoods** | **1.828** | **6.223** | **0.515, 3.140** | **0.006** |
| Other | -0.050 | 0.951 | -1.21, 1.115 | 0.933 |
| *Social opportunity* | | | | |
| **Sport & active recreation** | **1.316** | **3.728** | **0.352, 2.279** | **0.007** |
| **Education** | **2.134** | **8.450** | **0.776, 3.491** | **0.002** |
| Environment | 0.863 | 2.371 | -0.14, 1.871 | 0.093 |
| Neighbourhoods | 0.320 | 1.377 | -1.04, 1.688 | 0.647 |
| Other | -0.575 | 0.563 | -1.80, 0.654 | 0.359 |
| *Physical opportunity* | | | | |
| **Sport & active recreation** | **1.477** | **4.382** | **0.511, 2.443** | **0.003** |
| **Education** | **1.776** | **5.908** | **0.473, 3.079** | **0.008** |
| **Environment** | **1.135** | **3.110** | **0.104, 2.165** | **0.031** |
| Neighbourhoods | 1.091 | 2.978 | -0.28, 2.472 | 0.121 |
| Other | -0.280 | 0.756 | -1.49, 0.936 | 0.652 |
| *Reflective motivation* | | | | |
| **Sport & active recreation** | **2.066** | **7.896** | **1.127, 3.005** | **0.000** |
| **Education** | **1.939** | **6.950** | **0.622, 3.254** | **0.004** |
| **Environment** | **1.570** | **4.808** | **0.578, 2.562** | **0.002** |
| **Neighbourhoods** | **1.397** | **4.041** | **0.134, 2.658** | **0.03** |
| Other | -0.580 | 0.560 | -1.76, 0.600 | 0.336 |
| *Automatic motivation* | | | | |
| **Sport & active recreation** | **1.672** | **5.322** | **0.738, 2.604** | **<0.001** |
| **Education** | **2.114** | **8.281** | **0.721, 3.506** | **0.003** |
| **Environment** | **1.470** | **4.348** | **0.474, 2.464** | **0.004** |
| Neighbourhoods | 1.127 | 3.087 | -0.10, 2.364 | 0.074 |
| Other | -0.254 | 0.776 | -1.47, 0.968 | 0.684 |
| Health sector employment set as reference;Bold = significant at alpha 0.05. | | | | |

**Figures**


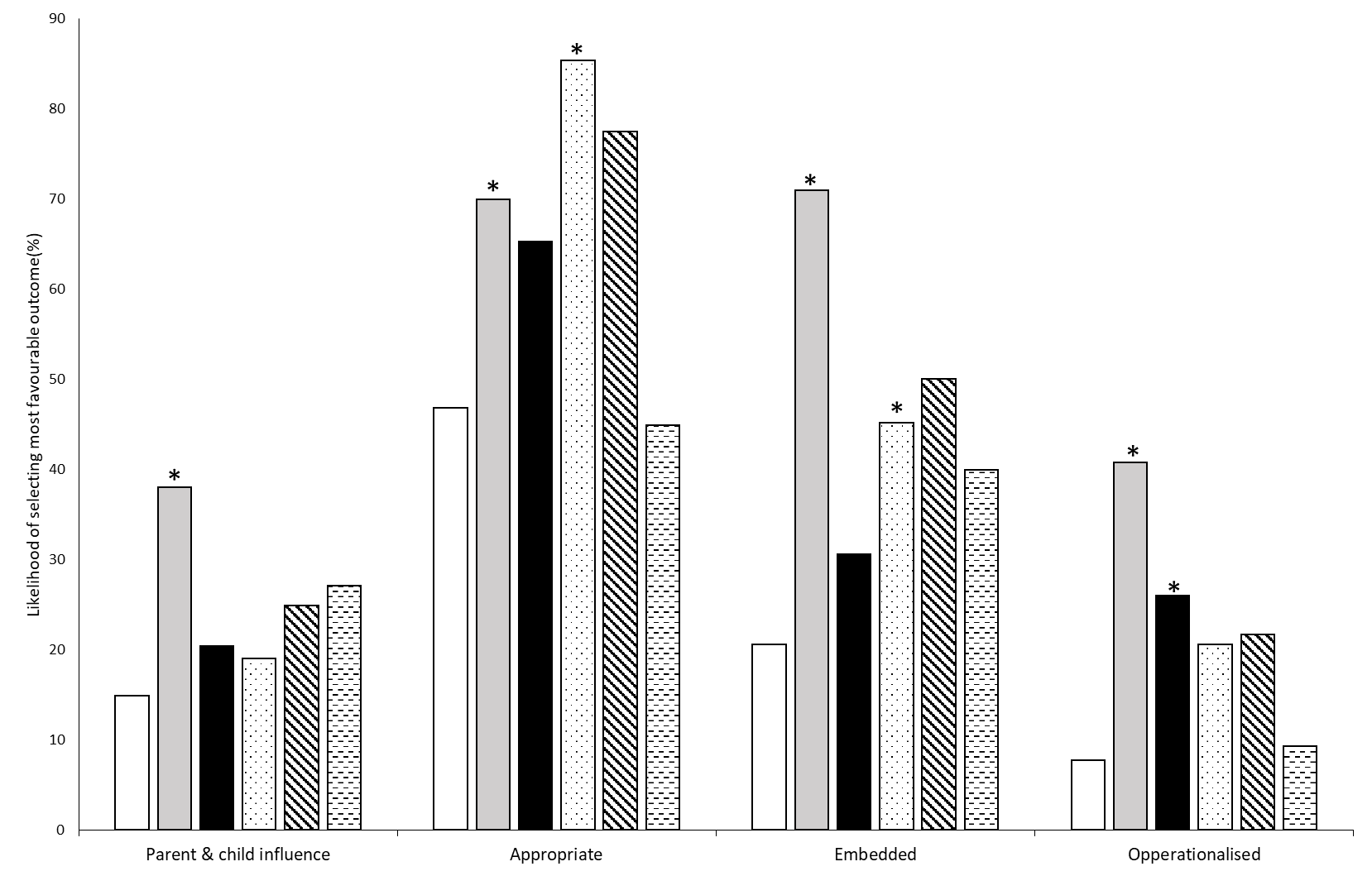


**Figure S1.** Likelihood of selecting the most favourable outcome for policy implementation by employment sector. Data presented as percentage (%,); * indicate significantly different to the reference group (health), alpha = 0.05. White = Health; Grey = Sport & active recreation; Black = Education; … = Environment; \\\ = Neighbourhoods; --- = Other.


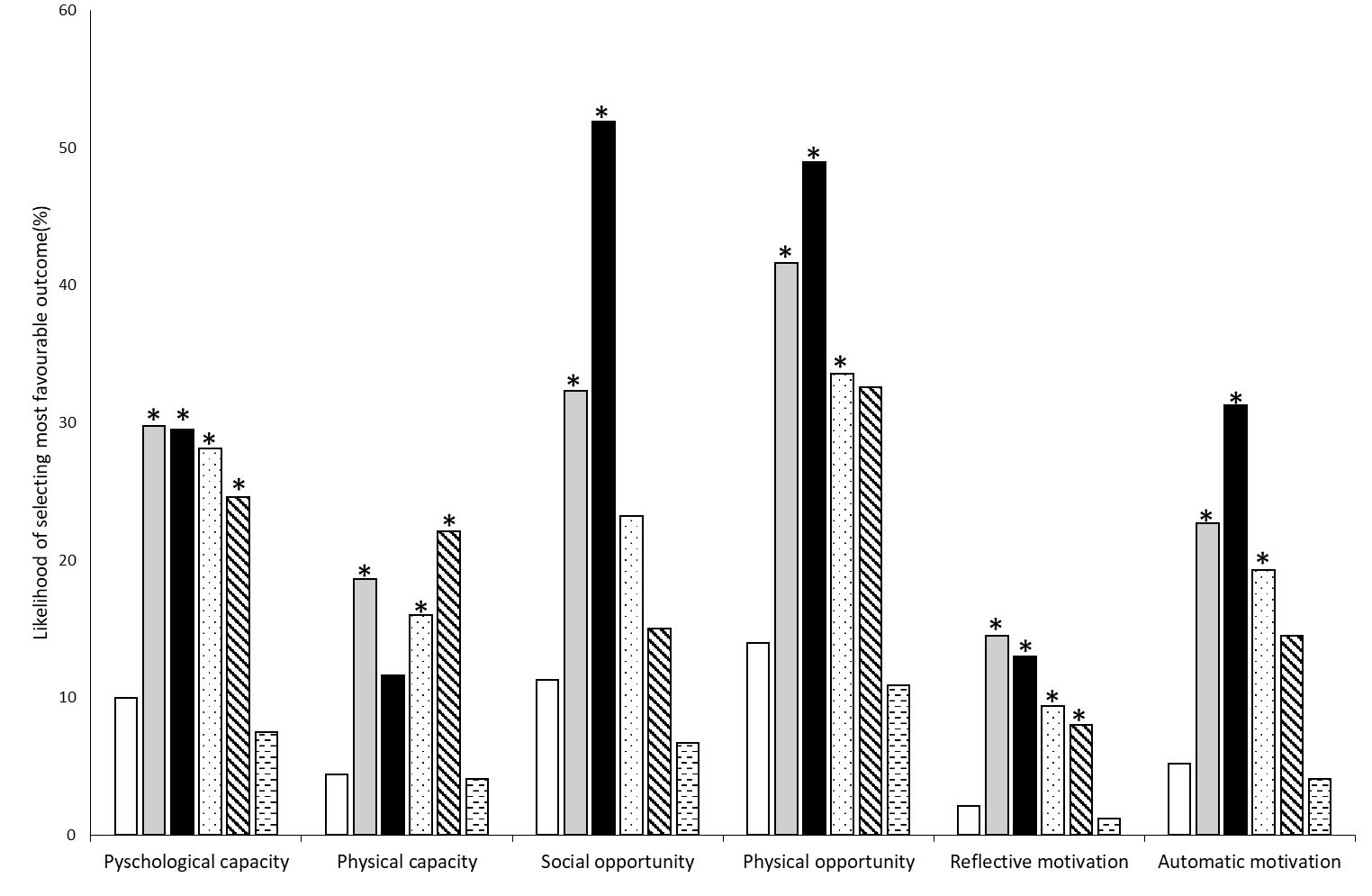


**Figure S2.** Likelihood of selecting the most favourable outcome for COM-B questions by employment sector. Data presented as percentage (%); * indicate significantly different to the reference group (health), alpha = 0.05. White = Health; Grey = Sport & active recreation; Black = Education; … = Environment; \\\ = Neighbourhoods; --- = Other.
